# Elucidating the mechanism of stigmasterol in acute pancreatitis treatment: insights from network pharmacology and *in vitro*/*in vivo* experiments

**DOI:** 10.3389/fphar.2024.1485915

**Published:** 2024-12-23

**Authors:** Xuanlin Zhao, Fan Li, Ao Wen, Xiuxian Yu, Xinrui Xu, Chengyu Wan, Yu Cao, Guang Xin, Wen Huang

**Affiliations:** ^1^ West China Center of Excellence for Pancreatitis, Institute of Integrated Traditional Chinese and Western Medicine, Natural and Biomimetic Medicine Research Center, Tissue-Orientated Property of Chinese Medicine Key Laboratory of Sichuan Province, West China School of Medicine, West China Hospital, Sichuan University, Chengdu, China; ^2^ Department of Emergency Medicine, West China School of Medicine, West China Hospital, Sichuan University, Chengdu, China

**Keywords:** stigmasterol, acute pancreatitis, apoptosis, network pharmacology, molecular docking

## Abstract

**Introduction:**

Acute pancreatitis (AP) is a severe inflammatory disease of the pancreas that could trigger a systemic inflammation and multi-organ dysfunction. Stigmasterol, a natural plant sterol found in various herbs and vegetables, exhibits a significant anti-inflammatory, antioxidant, and cholesterol-lowering effects. However, its therapeutic potential in AP have not been thoroughly investigated.

**Methods:**

The present study employed network pharmacology combined with experimental verification to explore the protective effect of stigmasterol on AP and its molecular mechanism in a sodium taurocholate (STC)-induced AP mouse model.

**Results:**

Protein-protein interaction (PPI) analysis pinpointed out MAPK3, also named as ERK1, as a promising stigmasterol target in AP therapy. Molecular docking analysis further revealed a strong binding capacity of stigmasterol to ERK1 (−6.57 kL/mol). Furthermore, both *in vivo* and *in vitro* studies demonstrated that stigmasterol treatment notably attenuated STC-induced pancreatic injury, as evidented by decreased serum levels of lipase and amylase, improved systemic inflammation, and reduced acinar cell necrosis. At the molecular level, stigmasterol treatment exhibited a significant inhibition on STC-induced activation of ERK signaling pathway in pancreatic acinar cells, leading to the transition of acinar cell death from necrosis to apoptosis, thereby preventing acinar cell necrosis-induced systemic inflammation.

**Conclusion:**

This study demonstrated that stigmasterol exhibits a significant protective effect aganist AP, at least in part through enhancing acinar cell apoptosis via modulating the ERK signaling pathways.

## 1 Introduction

Acute pancreatitis (AP) is a potentially life-threatening condition characterized by acute inflammation of the pancreas (Xiao et al., 2016; Dambrauskas et al., 2010; Akinosoglou and Gogos, 2014), often accompanied by a systemic inflammatory response and, in severe cases, multi-organ dysfunction (Mederos et al., 2021; Mayerle et al., 2019). The pathophysiology of AP is complex, involving a cascade of events that begin with the activation of digestive enzymes within the pancreas, leading to autodigestion and subsequent inflammation. Nevertheless, the AP pathogenesis remains elusive, necessitating further clarification. The outcome of AP is determined by the type of acinar cell death. The type of cell death including inflammatory death (ferroptosis, pyroptosis) and non-inflammatory death (apoptosis, autophagy) (Mayerle et al., 2019) Previous studies have confirmed that apoptosis plays a role of self-protection in pancreatitis, mainly due to the fact that apoptosis activation can inhibit other inflammatory death processes and thus avoid the occurrence of systemic inflammation, but the specific mechanism of action is still unclear (Kaiser et al., 1995; Bhatia et al., 1998; Chen et al., 2019). Therefore, it is important to explore the mechanism of inflammatory response and acinar cells apoptosis.

Natural products, including plant-derived compounds, have long been recognized as sources of potential therapeutic agents due to their diverse biological activities and relatively low toxicity (Ma et al., 2011; Zhang et al., 2021), providing numerous new avenues for AP treatment. Phytosterols, naturally occurring steroids abundant in plant tissues, are renowned for their health-bolstering attributes. Consumption of phytosterol-rich diets curbs cardiovascular risk and displays potent anti-inflammatory (Garcia-Llatas and Rodriguez-Estrada, 2011; Moreau et al., 2002) and antioxidant capacities effects (Moreau et al., 2002; Ramu et al., 2016). Stigmasterol (Stigma), a natural plant sterol widely distributed in various herbs and vegetables (Ramu et al., 2016; Aboobucker and Suza, 2019), has garnered attention for its anti-inflammatory, antioxidant, and cholesterol-lowering properties (Liang et al., 2020; Yuan et al., 2019; Sun et al., 2019; Zhang et al., 2023). The multifaceted nature of stigmasterol’s biological activities suggests that it may have potential in the treatment of inflammatory diseases, including AP. Recent studies show that the anti-oxidation and anti-inflammatory effects of Stigma may be related to its regulation of apoptosis (Lu et al., 2023), such as induced the activity of apoptotic proteins, including cleaved caspase-3, cleaved caspase-9, cytochrome c, and BAX. In view of the self-protective role of apoptosis in the progression of pancreatitis, we hypothesize that Stigma might promote the transformation of pancreatic acinar cells from necrosis to modulation, providing therapeutic potential in AP.

This study aims to explore the potential of stigmasterol in the context of AP using a combination of network pharmacology and experimental validation. Network pharmacology is an integrative approach that applies systems biology principles to analyze the interactions between drugs and biological systems, offering a comprehensive understanding of drug action (Xiong et al., 2020; Huang et al., 2017). This methodology is particularly well-suited for complex diseases like AP, where multiple pathways and targets are implicated in the disease process (Feng et al., 2024; Zhang et al., 2024). Specifically, we employed PPI analysis to identify potential stigmasterol targets in AP and then conducted molecular docking analysis to predict the binding affinity of stigmasterol to these targets. Using PPI and molecular docking, we found that MAPK3 (ERK1) is an effective target for the treatment of acute pancreatitis. Extracellular signal-regulated kinase 1 (ERK1) has been shown to play a crucial role in the pathogenesis of AP. As a member of the MAPK family, ERK1 is involved in cell proliferation, differentiation, and apoptosis. Modulating MAPK signaling pathways has been shown to promote the apoptosis of acinar cells while reducing inflammatory damage to the pancreas. However, it is unclear whether Stigma treatment would affect the ERK1 signaling pathway and result in the modulation of acinar cell apoptosis.

**FIGURE 1:**
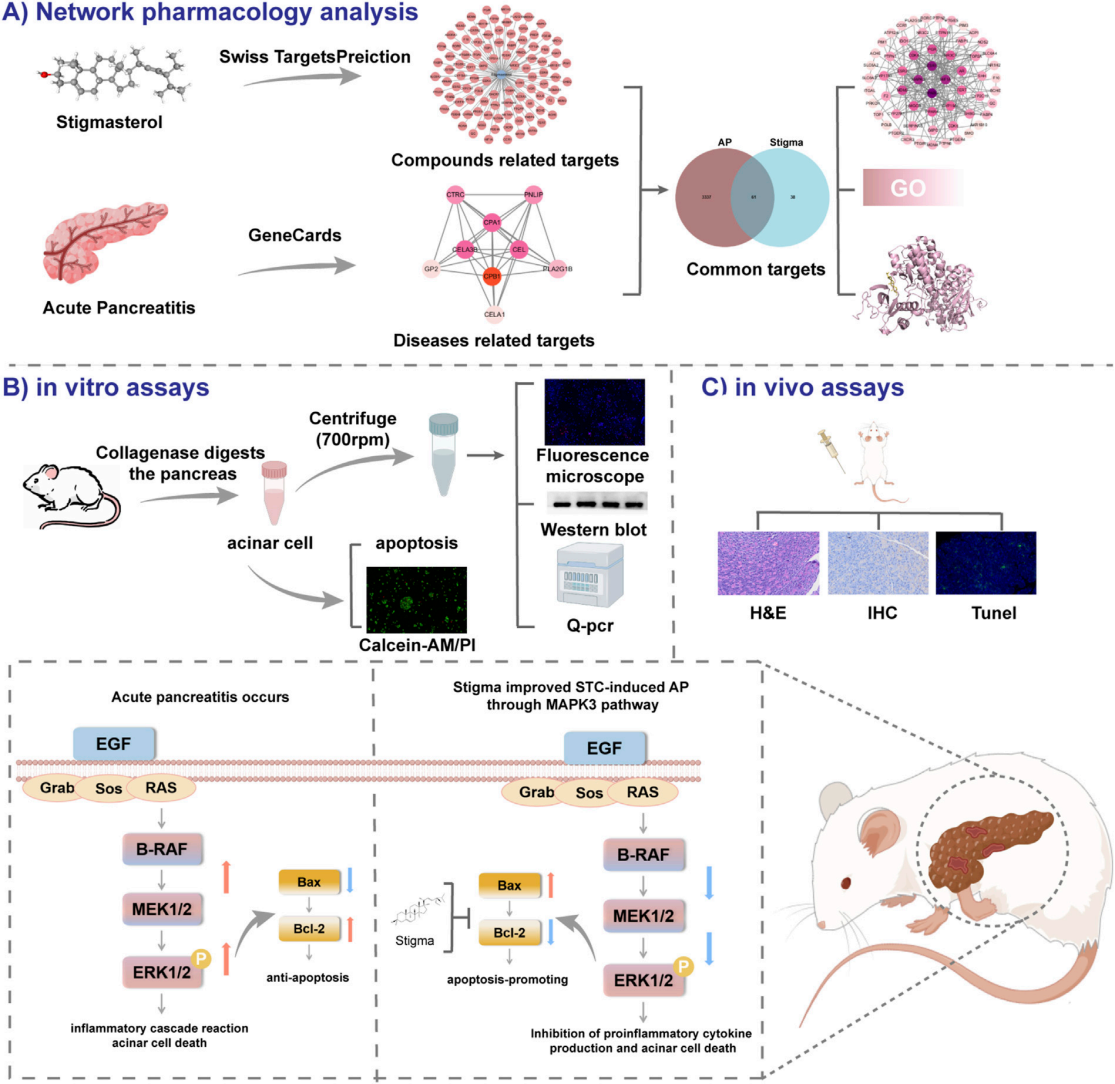
All technical approaches rely on network pharmacology and experimental verification. **(A)** Network Pharmacology Analysis. **(B)** *in vitro* assays. **(C)** *in vivo* assays.

In this study we hypothesized that Stigma treatment could protect mice from pancreatic injury through enhancing cell apoptosis via modulating ERK1 signaling pathway. To address this hypothesis, we evaluated changes in pancreatic histology, serum amylase and lipase activities to elucidate the impact of Stigma on AP-induced damage at both cellular and systemic levels. Moreover, Western blotting, Immunohistochemical, and TUNEL Staining were conducted to determine the regulatory role of Stigma on cell apoptosis and ERK1 signaling pathway. The research route is illustrated in Figure 1.

## 2 Materials and methods

### 2.1 Chemicals and reagents

Stigmasterol (HY-N0131) were acquired from MCE (Shanghai, China). Cell counting kit-8 (MA0218-1) and Propidium Iodide (MB2920-1) was obtained from meilunbio (Dalian, China). Hoechst 33342 (40731ES10) were purchased from Yeasen Biotechnology Co, Ltd. (Shanghai, China). Calcein AM (C2012) was obtained from Beyotime Biotechnology (Shanghai, China). DeadEnd™ Fluorometric TUNEL System (G3250) were purchased from Promega (America).

### 2.2 Data collection

The PubChem database furnished the generic name, CID, and 3D configuration of Stigma. SwissTargetPrediction database contributed Stigma-derived target sets, while GeneCards sourced AP-associated targets. Commonality analysis among these targets was executed utilizing Venny 2.1.0 software.

### 2.3 Network analysis

Network analyses were conducted with Cytoscape 3.9.1, where nodes’ significance was quantified by scores, inversely correlated with color intensity. STRING facilitated the construction of a PPI network for shared targets, revealing interconnected target clusters. Within these clusters, the most significant node, designated SEED, emerged as a potential cluster key target. Consequently, the top 15 genes and SEEDs were prioritized as crucial hubs from the PPI landscape.

### 2.4 Gene ontology enrichment analysis

We employed the KOBAS 3.0 platform to execute GO enrichment investigations on shared targets and hub genes, applying a p-value threshold of <0.05 for result filtration. The top 15 enriched GO terms were visually represented using Omicshare via circular, histogram, and bubble diagrams.

### 2.5 Molecular docking

The RSCB PDB database facilitated the retrieval of gene structures, while PubChem sourced the SDF format of compounds. PyMOL software was instrumental in docking, visualizing protein residues and binding interactions, creating 3D structures, and displaying the intricate molecular interactions.

### 2.6 Experimental animals

Ethical clearance for the study’s entirety was granted by the Ethics Review Board of West China Hospital, Sichuan University (Approval ID: 20220221068). Male C57BL/6 mice, weighing 23–25 g and aged 6–8 weeks, were procured from SPF Biotechnology Co., Ltd. (Chengdu, China). The mice underwent a week-long acclimatization period in a pathogen-free environment, with temperature regulated at 20°C–22°C, humidity maintained at 55%, and a standard 12-h light-dark cycle.

### 2.7 AP model establishment and experimental design

Prior to surgery, animals underwent a 12 h fasting period. Mice were randomly divided into four groups, each containing 5-8 animals. STC-induced AP mouse model: After anesthesia with 1% pentobarbital sodium, AP was induced through retrograde injection into the pancreatic duct using 3.5% sodium taurocholate (STC). Stigma was administered intraperitoneally at doses of 50 mg/kg or 100 mg/kg, with saline serving as the control, given 1 hour after model induction. Stigma was dissolved in saline. 24 h after administration, mice were anesthetized with an intraperitoneal injection of 1% pentobarbital, then euthanized by cervical dislocation, and serum, pancreas, and lung tissues were collected.

### 2.8 Primary pancreatic acinar cells isolation

Primary pancreatic acinar cell are prepared with modification of the technique developed by Delia Menozzi (Menozzi et al., 1990) and Zhao et al. (Zhao et al., 2018). Fresh pancreas was infused with buffer A (140 mM NaCl, 4.7 mM KCl, 1.13 mM MgCl_2_, 1 mM CaCl_2_, 10 mM glucose, 10 mM HEPES, and 0.5 mg/mL soybean trypsin inhibitor [pH 7.3]) containing 0.3 mg/mL collagenase, minced and digested (19 min, 100 rpm/min shaking, 37°C). Mechanical disruption separated the cells, which were then passed through a 100-μm cell strainer. Cell precipitation was achieved by centrifugation at 700 rpm for 2 minutes, and the cell pellet was resuspended in HEPES buffer, and complete the follow-up experiment within 5 hours.

### 2.9 Histological examination

Pancreatic tissues were fixed in 10% formalin, dehydrated and embedded in paraffin. Tissue sections were cut and stained with hematoxylin and eosin (H&E) for histological examination.

### 2.10 TUNEL staining

Pancreatic tissues were fixed in 10% formalin, dehydrated, and paraffin embedded. Tissue sections were excised, followed by TUNEL staining. The specific methods are as follows (Kyrylkova et al., 2012): 1) Wash sections 2× with PBS, 5 min each time (to remove OCT); 2) Fix sections with 4% paraformaldehyde in PBS for 15 min; 3) Wash the sections 2× with PBS, 5 min each time; 4) Treat sections with 1 μg/mL proteinase K in PBS for 10–20 min; 5) Wash sections with PBS for 5 min; 6) Refix sections with 4% paraformaldehyde in PBS for 5 min; 7) Wash sections 2× with PBS, 5 min each time; 8) Prepare a positive control; 9) Remove excess liquid from the slides. Add 100 μL per slide of equilibration buffer. Cover slides with plastic coverslips -carefully and incubate for 5–10 min; 10) Carefully remove plastic coverslips and excess buffer from the slides. Add 100 μL per slide of TdT reaction mix. Meanwhile, prepare a negative control (optional): Add -negative control mix instead of TdT reaction mix. Cover the slides with plastic coverslips carefully. Incubate the slides at 37°C for 60 min; 11) Wash sections with 2× SSC for 15 min; 12) Wash sections 3× with PBS, 5 min each time; 13) Add 120 μL per slide of SA–Cy3 diluted in PBS (1:500). Cover the slides with plastic coverslips carefully. Incubate the slides for 30–45 min; 14) Wash sections 3× with PBST, 10 min each time; 15) Transfer slides to a slide rack. Wash the sections 2× with dH2O, 3 min each time; 16) Dehydrate sections 1× in 50, 70, and 95% ethanol in water, and 2× in 100% ethanol, 3 min each time; 17) Incubate sections 2× in xylene, 3 min each time; 18) Mount slides with DPX and apply micro cover glasses, being careful not to trap any air bubbles. Let slides dry overnight.

### 2.11 Cell culture and cytotoxicity study

266–6 cell were cultured in Dulbecco’s modified Eagle’s medium (DMEM) with 10% fetal bovine serum (FBS) and 1% penicillin/streptomycin at 37°C and 5% CO2 humidity. Inoculation of 266–6 cell in 96-well plates at a concentration of approximately 1.5*104/100 μL per well (n = 3). 24 h later, the plates were incubated with different concentrations of Stigma (25, 50, 100, and 200 μM) in the well plates for 24 h. After 24 h, 10 μ of CCK-8 solution was added to each well. The cytotoxicity assay was measured with the Cell Counting Kit-8 (CCK-8) assay.

### 2.12 Serum amylase and lipase measurement

Blood collected post-anesthesia via cardiac puncture into serum separator tubes was centrifuged at 3,000 rpm for 10 min to obtain serum. A 50 μL aliquot of serum was diluted with distilled water to a final volume of 300 μL, and serum amylase and lipase levels were measured using a fully automated biochemical analyzer (Roche, Mannheim, Germany) following the manufacturer’s instructions.

### 2.13 Propidium iodide (PI) staining

Freshly isolated primary pancreatic acinar cells were treated with STC (final concentration 5 mM) and co-incubated with Stigma at 37°C for 50 min. Cells were subsequently stained with Hoechst 33342 (50 μg/mL) and propidium iodide (PI, 1 μmol/mL). Images were captured using an upright fluorescence microscope (Axio Imager Z2, Zeiss, Oberkochen, Germany), and displaying Hoechst 33342/PI fluorescence. Upon analyzing each stained preparation, 3 randomly selected fields of view per group were counted using ImageJ to count all live cells and all pi-positive (necrotic) cells in the area.

### 2.14 Microscale thermophoresis (MST) assays

The dye was first incubated (Entzian and Schubert, 2016) with ERK1 recombinant protein for 30 min and placed on ice to be assayed. A starting concentration of 100 μm was selected for Stigma, which was then 2-fold gradient diluted to 16 concentrations. 10 μL of 200 nM aptamer working solution was added to 10 μL of each ligand dilution and the samples were mixed by pipetting. Incubate the samples at room temperature for 5 min, then fill a standard capillary with the sample. Place the capillary on the capillary tray and insert it into the MST device.

### 2.15 Q-PCR analysis

The total RNA of was isolated using TRIzol™ reagent (Invitrogen, Carlsbad, CA, United States) according to the manufacturer’s instructions. Total RNA was reverse-transcribed using Hifair III first Strand cDNA Synthesis SuperMix (Yeasen, Shanghai, China). Real-time qPCR was performed using Hieff^®^ qPCR SYBR Green Master Mix Kit (Yeasen, Shanghai, China) on a Thermo Q6 Real-Time System, according to the manufacturer’s instructions. Gene expression relative to that of β-actin was analyzed for each sample using the 2^−ΔΔCT^ method. The primers were designed and synthesized as follows:

TNF-α-F: 5′-CCT​GTA​GCC​CAC​GTC​GTA​G-3′;
TNF-α-R: 5′-GGG​AGT​AGA​CAA​GGT​ACA​ACC​C-3′;
IL-1β-F: 5′-GAA​ATG​CCA​CCT​TTT​GAC​AGT​G-3′;
IL-1β-R:5′- TGG​ATG​CTC​TCA​TCA​GGA​CAG-3′;
IL-6-F: 5′-AGT​TGC​CTT​CTT​GGG​ACT​GA-3′;
IL-6-R:5′-TCCACGATTTCCCAGAGAAC-3′;
β-actin-F:5′-GTGACGTTGACATCCGTAAAGA-3′;
β-actin-R:5′-GCCGGACTCATCGTACTCC-3′.

### 2.16 Western blot analysis

Similar to the accepted experimental procedure, in simple terms, the protein was separated by SDS-PAGE gels, then transported to PVDF membrane, and finally incubated with primary antibody and HRPlabeled secondary antibody, and the final blot image was observed.

### 2.17 Statistical analysis

Data are presented as the mean ± standard deviation (SD). Statistical analysis were conducted using GraphPad Prism 8.0, employing two-sided Student’s unpaired t-tests or one-way analysis of variance (ANOVA) as appropriate. We did a G-power on all our results based on the sample sizes and test levels of the study results, which confirmed that all statistical power were greater than 0.8.

## 3 Results

### 3.1 Screening of potential targets

**FIGURE 2:**
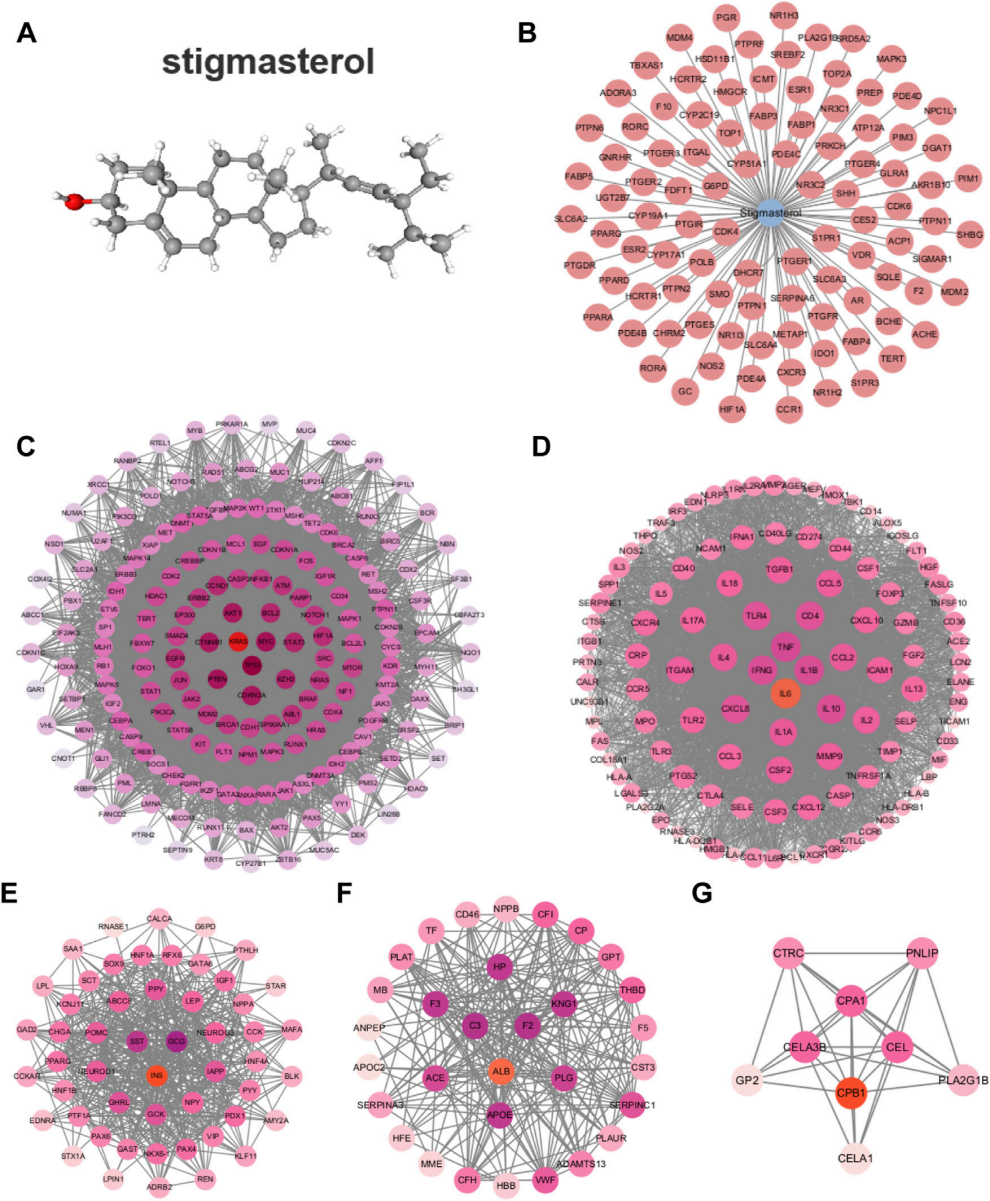
Network analysis of predict targets for Stigma compound and AP. **(A)** Structure of the Stigma. **(B)** Potential targets for Stigma. **(C–G)** Cluster analysis identified the top 5 core seed nodes involved in AP in the corresponding clusters. The seed nodes in each cluster were marked with red circles.

Stigma is a phytosterol with anti-inflammatory properties, its chemical structure formula is shown in Figure 2A (Haque et al., 2021). In this study, the SwissTargetPrediction database was utilized to predict targets for Stigma, and a total of 99 potential targets were obtained (Figure 2B). GeneCards was used to screen 3,398 potential targets which related to AP. Cluster analysis was conducted using the MCODE plug-in, yielding five clusters including KRAS, IL-6, INS, ALB, CPB1. Each cluster was marked by a red circle representing a seed node (Figures 2C–G).

### 3.2 Cluster analysis of PPI network with compound-disease common targets

**FIGURE 3:**
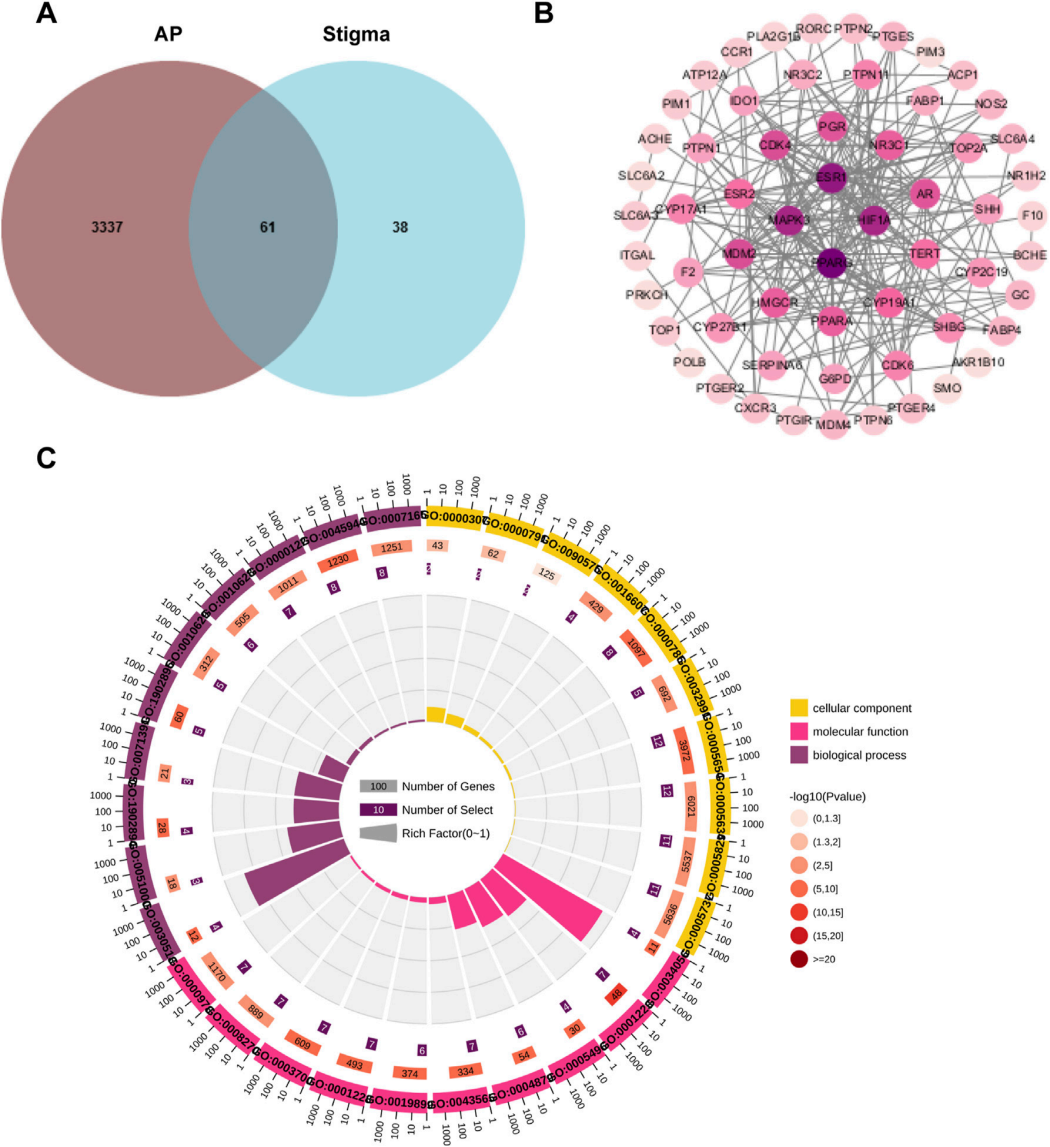
Cluster analysis of PPI network with compound-disease common targets. **(A)** Venny diagram of 61 targets, which was intersection of the Stigma compound predicted targets and AP-related targets. **(B)** The PPI network of 61 common targets. **(C)** Top 15 BP, CC and MF in GO analysis of the common 61 targets.

The Venny diagram showed that 61 common targets were identified by matching 99 drug targets and 3,398 disease targets (Figure 3A), which are potential therapeutic targets of Stigma for acute pancreatitis. The 61 targets are imported into the STRING database to obtain the PPI network. There are 61 Nodes and 221 Edges in the PPI network. The “Cytoscape” plug-in can be used to analyze the Degree value of the targets, and the analyzed topological parameters can be used to visualize the PPI network, the larger the shape and the darker the color, the larger the Degree value of the node (Figure 3B). The top 15 core targets were selected: PPARG, ESR1, HIF1A, MAPK3, MDM2, CDK4, PGR, AR, PPARA, HMGCR, NR3C1, CYP19A1, ESR2, TERT, CYP17A1.

GO analysis and KEGG analysis were performed on the intersecting targets, including biological processes, cellular components and molecular functions, The enrich circle plot were used to visualize the enrichment results of the top 15 GO terms. The outermost circle showed the classification of GO enrichment, and the purple represents BP, the pink represents MF, the yellow represents CC. The second circle showed the P-value, the smaller the value, the darker the color. The third circle showed the total number of foreground genes. The fourth circle showed the RichFactor value of each classification. The results showed that BP was most related to positive regulation of nitric-oxide synthase activity, CC was most related to RNA polymerase II transcription factor complex, and MF was most related to RNA polymerase II core promoter proximal region sequence-specific DNA binding (Figure 3C).

### 3.3 The binding studies of ERK1 and stigma

**FIGURE 4:**
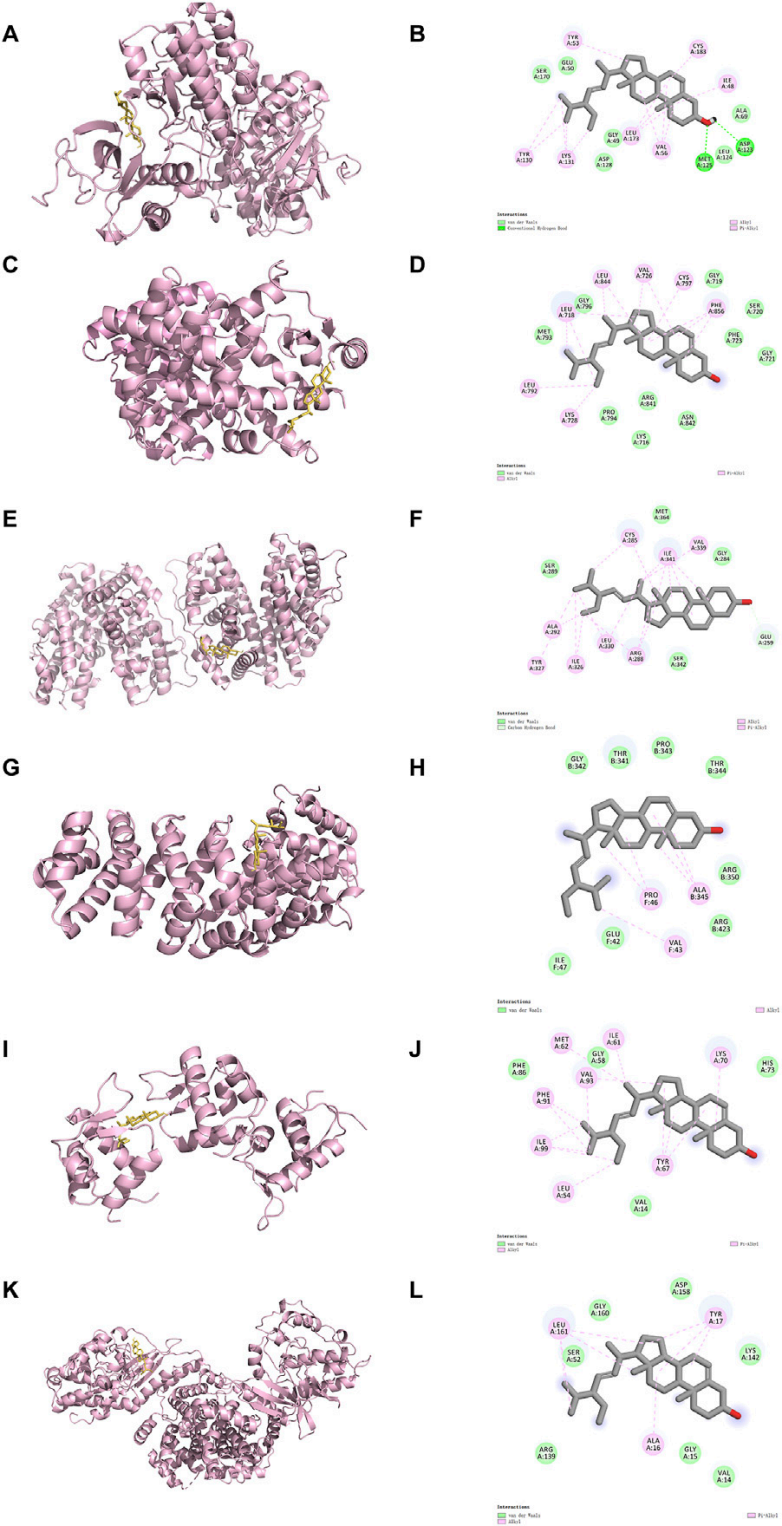
Results of molecular docking and the 2D docking diagram of Stigma. **(A, B)** MAPK3, **(C, D)** EGFR, **(E, F)** PPARG, **(G, H)** HIF1A, **(I, J)** MDM2 and **(K, L)** CDK4.

We next select the top 6 targets of Stigma to conduct molecular docking analysis to confirm the interaction between Stigma and its potential targets. A reduced docking score correlates positively with enhanced ligand-receptor binding strength, predictive of heightened interaction potential. Binding energies for all targets against Stigma fell below −5.0 kJ/mol. MAPK3 was rated as low as −6.57, suggesting which may be an important target for Stigma in the treatment of AP. 3D binding configurations are visualized in Figure 4, revealing probable intermolecular engagements between core compounds and their protein targets, as depicted in the docking analyses. Furthermore, we introduce microscale thermophoresis (MST) as a tool to characterize protein-small molecule interactions in biological liquids. Notably, the results of MST assays indicate that ERK1 and Stigma may directly interact with each other (Supplementary Figure S1). Taken together with other data, these results suggest that Stigma may exert its pharmacological activity through ERK1.

### 3.4 Stigma treatment relieved the severity of STC-induced AP in mice

**FIGURE 5:**
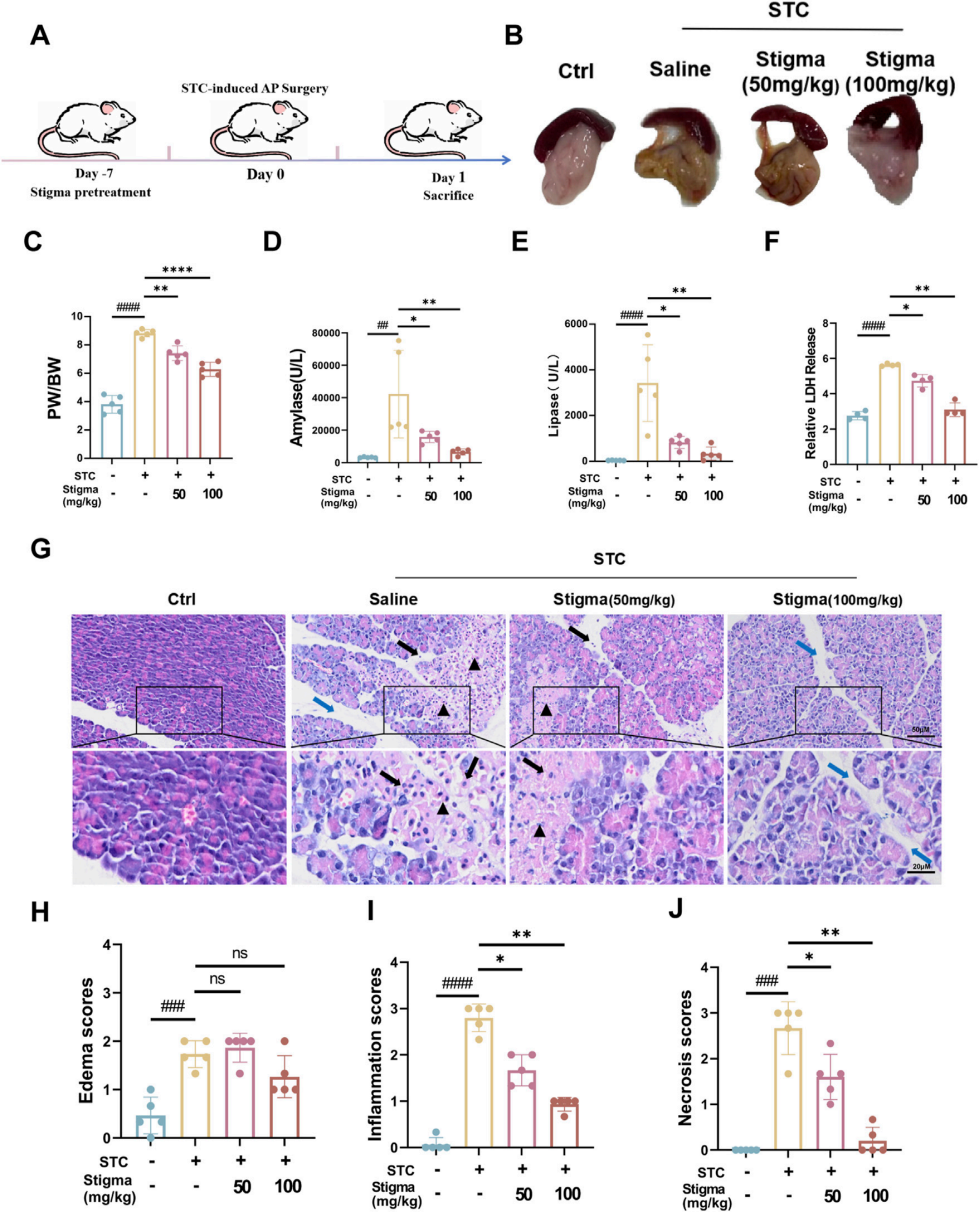
Stigma relieves STC-induced AP in mice. **(A)** Schematic diagram of the establishment of STC-induced AP mouse model and the therapeutic process via i v. (intravenous) injection. **(B)** Representative images of pancreatic morphology. **(C)** Ratio of pancreas to body weight in mice. **(D, E)** Serum levels of amylase and lipase in mice. **(F)** LDH Release levels in mouse serum. **(G)** H&E staining of mice pancreatic sections. Stained sections were taken at magnification of 50 μm and 20 μm. **(H–J)** Histopathological scoring of pancreatic tissue involving three assessment indices: edema, inflammatory infiltration and parenchymal necrosis. Black arrows point to inflammatory cells, triangles mark areas of necrosis, and blue arrows point to the width of the pancreatic lobular space, representing the degree of edema. All data are expressed as mean ± SD. n = 5. All the *p < 0.05. ns: not significant.

To verify the therapeutic activity of Stigma *in vivo*, we established a STC-induced AP mouse model and treated the mice according to the drug regimen designed in Figure 5A. The results indicated that the pancreatic tissues of mice in the AP group exhibited obvious edema, inflammatory infiltration. Notably, compared to the AP group, both 50 mg/kg and 100 mg/kg Stigma ameliorated the pancreatic tissue pathological damage, with the higher dose demonstrating a more pronounced improvement effect (Figure 5B). We statistically analyzed the ratio of pancreatic weight to body weight to assess the degree of pancreatic edema in each group of mice, and the results were shown in Figure 5C. The pancreas/body weight ratio was significantly higher in the STC group compared with that of the Sham group, and the pancreas/body weight ratio in the 100 mg/kg Stigma group was significantly lower than that of the STC group. Furthermore, serum lipase and amylase levels were found to be significantly elevated in STC mice, while treatment of Stigma reduced these indicators, with the 100 mg/kg Stigma group exhibiting a more significant therapeutic effect (Figures 5D, E). Moreover, Stigma intervention markedly decreased LDH leakage into the serum of AP mice subjected to STC challenge, indicative of its protective role in acinar cell necrosis (Figure 5F). Notably, H&E staining revealed that Stigma administered intraperitoneally mitigated STC-induced pancreatic tissue injury, including edema, inflammatory cell infiltration, and acinar necrosis, with the 100 mg/kg dose yielding a superior therapeutic response (Figures 5G–J). Collectively, these findings underscore the protective potential of Stigma against STC-induced AP in mice.

### 3.5 Stigma reduces inflammation in mice with AP

**FIGURE 6:**
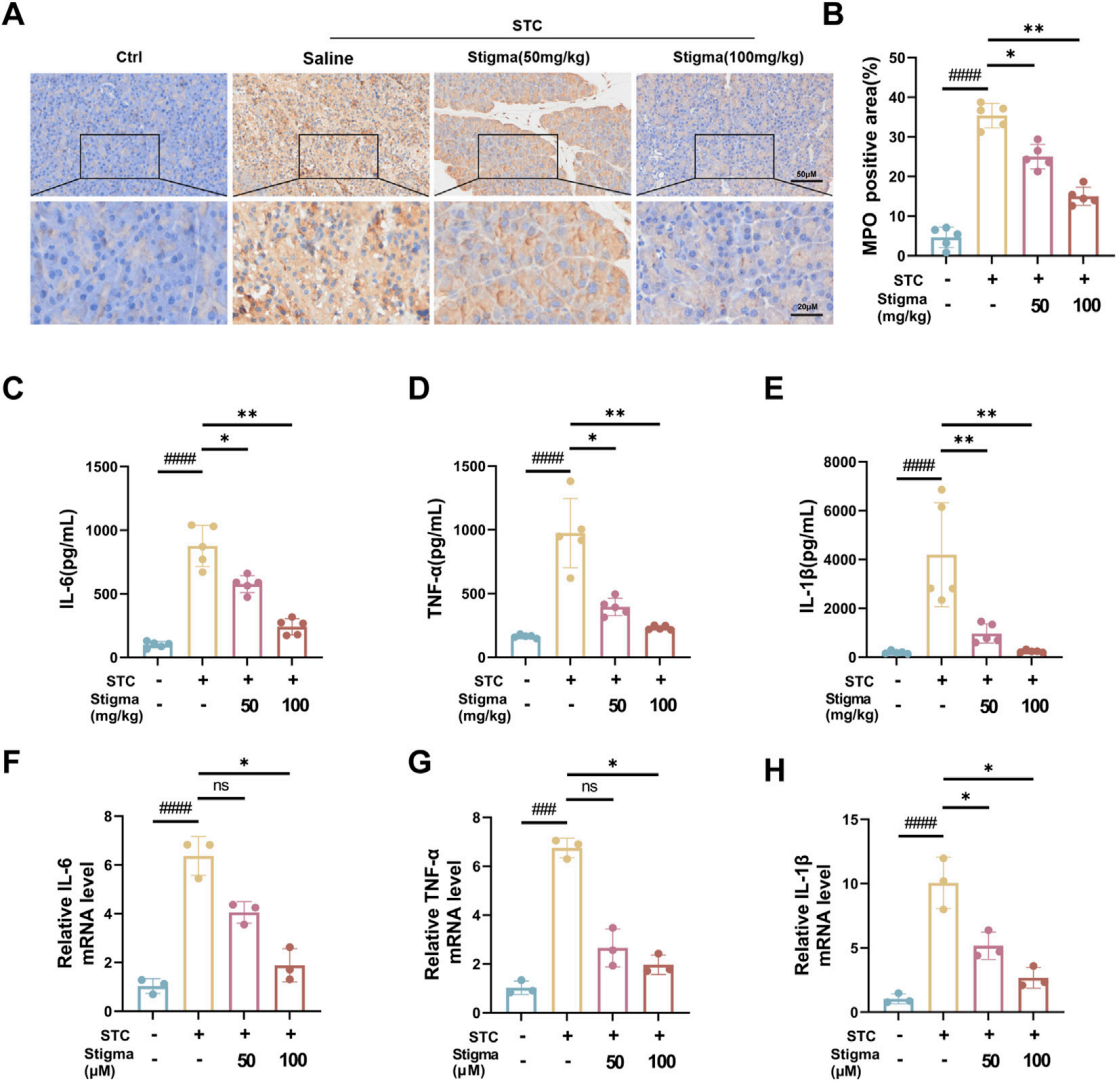
Stigma reduces inflammation in AP mice. **(A, B)** Representative immunohistochemistry images and quantitative analysis of MPO on the pancreas sections in STC-induced AP model mice treated with or without Stigma (i.g, 50 or 100 mg/kg/d, 7d). Stained sections were taken at magnification of 50 μm and 20 μm. **(C–E)** Levels of IL-6, TNF-α and IL-1β in mouse serum were determined by ELISA. **(F–H)** IL-6, TNF-α and IL-1β levels in STC stimulated primary pancreatic acinar cells were measured by quantitative PCR assay. All data are expressed as mean ± SD. n = 3 or 5. All the *p < 0.05. ns: not significant.

Myeloperoxidase (MPO), an established marker of neutrophil activation, offers valuable insights into neutrophil infiltration assessment. Immunohistochemical (IHC) analysis was employed to quantify MPO expressions. Notably, strong MPO immunostaining was discernible in pancreatic acinar cell of the AP group. Stigma, administered at varying concentrations, significantly attenuated MPO levels, with the 100 mg/kg dose exhibiting superior efficacy over 50 mg/kg (Figures 6A, B). This finding aligns with HE staining outcomes, suggesting that Stigma mitigates acinar cell damage in STC-induced acute pancreatitis. Cytokine-driven inflammatory responses, including those mediated by tumor necrosis factor-α (TNF-α), IL-1β, and IL-6, play a pivotal role in the progression of AP (Papachristou, 2008). As shown in Figures 6C–E, the AP group showed significantly raised levels of serum TNF-α, IL-6, and IL-1β in comparison with the sham group. To determine whether Stigma treatment could downregulate the transcription levels of inflammatory cytokines in STC-induced acute pancreatitis, we investigated these inflammatory cytokines using q-PCR. In the AP groups, the levels of TNF-α, IL-6 and IL-1β was significantly increased, whereas Stigma treatment reduced their production (Figures 6F–H). These results demonstrate that Stigma treatment effectively mitigates the production of inflammatory cytokines in STC-induced acute pancreatitis.

### 3.6 Stigma inhibits STC-induced AP through ERK1 pathway

**FIGURE 7:**
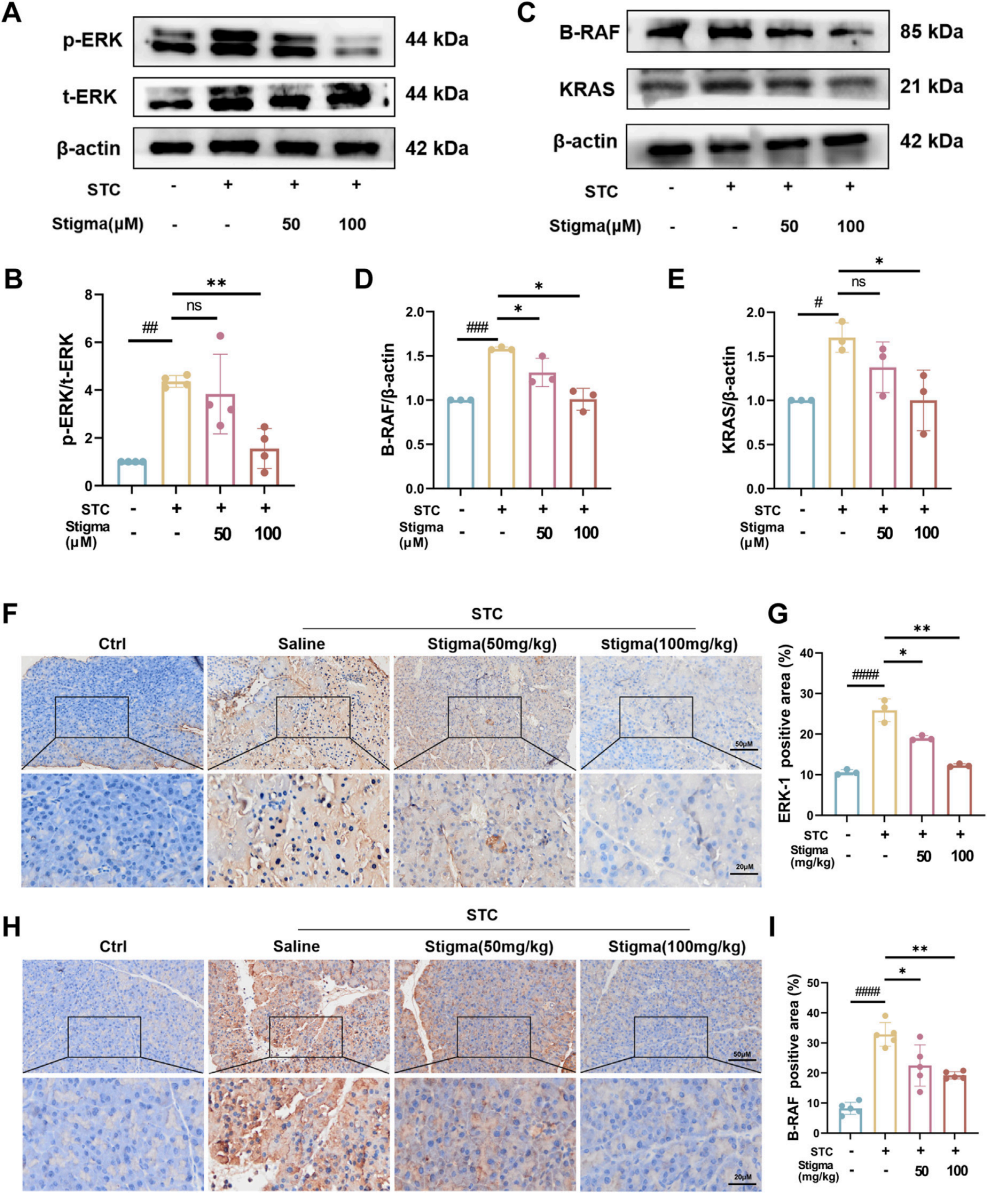
Stigma inhibits p-ERK/B-RAF/KRAS pathway with STC-induced AP in mice. **(A, B)** Representative Western blotting and quantitative analysis of p-ERK proteins in primary pancreatic acinar cells. **(C–E)** Representative Western blotting and quantitative analysis of B-RAF and KRAS proteins in primary pancreatic acinar cells. **(F, G)** Representative immunohistochemistry images and quantitative analysis of p-ERK on the pancreas sections in sodium taurocholate (STC)-induced AP model mice treated with or without Stigma (i.g, 50 or 100 mg/kg/d, 7d) **(G–I)** Representative immunohistochemistry images and quantitative analysis of B-RAF and KRAS on the pancreas sections in sodium taurocholate (STC)-induced AP model mice treated with or without Stigma (i.g, 50 or 100 mg/kg/d, 7d). Stained sections were taken at magnification of 50 μm and 20 μm. All data are expressed as mean ± SD. n = 3 or 5. All the *p < 0.05. ns: not significant.

Based on the hub targets and pathway predictions from network pharmacology, the therapeutic effect of Stigma appears to be associated with ERK1 signaling pathway. To validate these results, we performed Western blotting (WB) analysis, which revealed a significant increase in the expression of p-ERK in pancreatic acinar cell stimulated by STC (Figure 7A). Moreover, Stigma mitigated the upregulation of p-ERK expression observed in STC-induced AP, consistent with the results derived from network pharmacology (Figure 7B), indicating that ERK related pathways are crucial for the therapeutic effect of Stigma in AP. Subsequently, leveraging the results from Cluster analysis and KEGG, we performed Western blotting analysis on the upstream and downstream proteins of p-ERK in the AP pathway. The results showed that the expression of B-RAF and KRAS was upregulated in STC-induced AP, but this upregulation was reversed by Stigma treatment (Figures 7C–E). To deepen our understanding of the connection between STC-induced AP and the ERK1 signaling cascade, we delved into the expressions of p-ERK and B-RAF via IHC analysis. Our findings indicated an increase in the IHC staining for p-ERK and B-RAF in the AP group. In contrast, Stigma intervention mitigated this positivity, with a more discernible decline noted in the high-dose regimen. (Figures 7F–I).

Collectively, these results demonstrate that Stigma mitigates acinar cell injury in STC-induced AP may be by modulating ERK1-related pathways.

### 3.7 Stigma relieves primary pancreatic acinar cells injury in STC-induced AP

**FIGURE 8:**
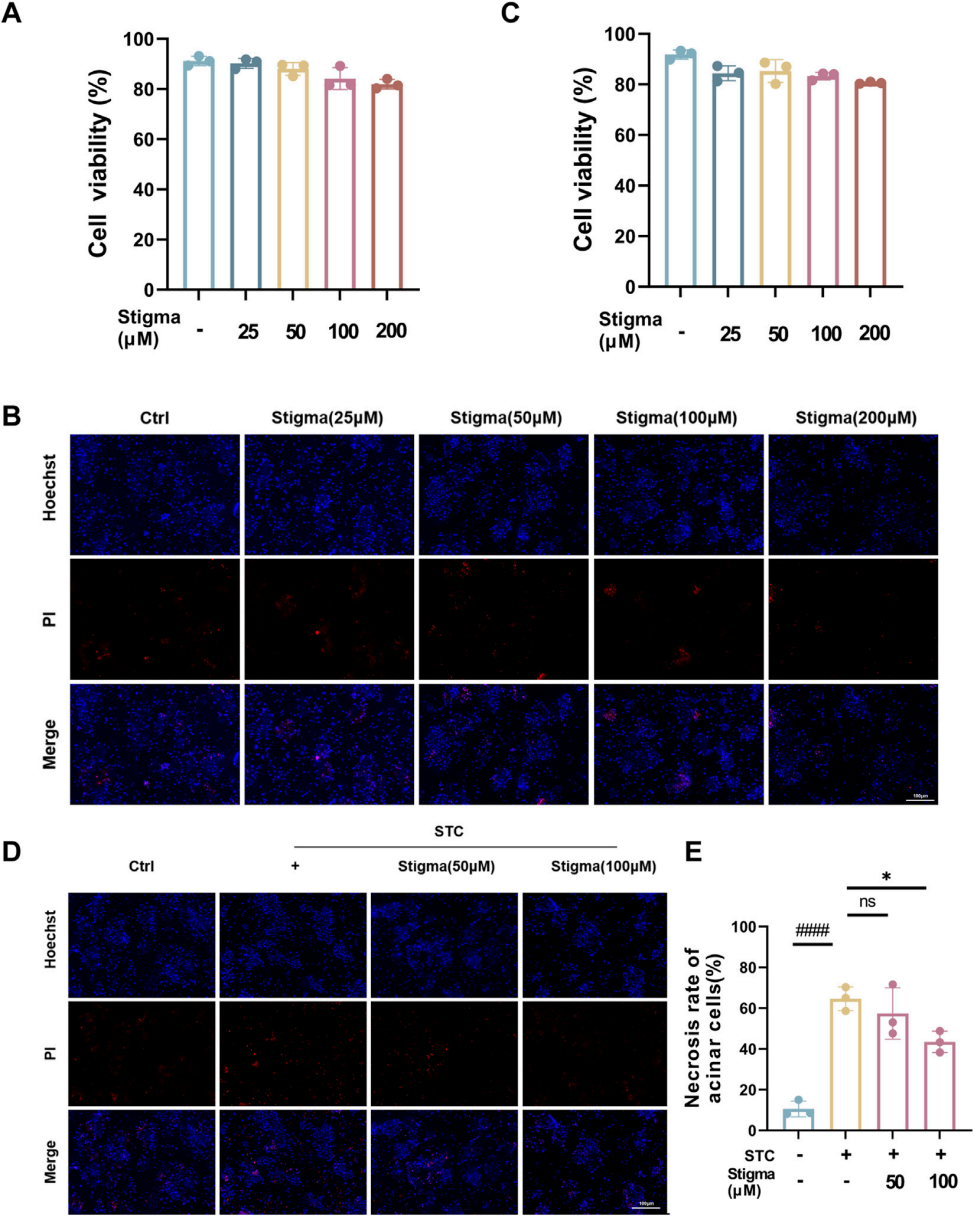
Stigma relieves primary pancreatic acinar cells injury in STC-induced AP. **(A)** The cytotoxicity of Stigma is evaluated with CCK-8 assay. **(B, C)** Representative images and quantitative analysis of Hoechst 33,342 and PI staining in primary pancreatic acinar cells. 1 - (number of PI stained cells (necrotic) divided by number of Hoechst 33,342 positive cells) to calculate cell viability percentage (%), 100 μm scale bar. **(D)** Representative images of Hoechst 33,342 and PI staining in STC stimulated primary pancreatic acinar cells treated with or without Stigma. 100 μm scale bar. **(E)** Quantitative analysis of STC-induced primary pancreatic acinar cell necrosis. The number of PI-stained cells (necrotic) was divided by the number of Hoechst 33,342-positive cells to calculate the percentage of necrosis (%). All data are expressed as mean ± SD. n = 3. All the *p < 0.05. ns: not significant.

In deeply explore the potential protective effect of Stigma in STC-induced necrosis in primary pancreatic acinar cell, we first examined the cytotoxicity of various concentrations of Stigma using the CCK-8 assay kit, along with Hoechst and PI live-dead cell staining. The CCK-8 assay (Figure 8A) and Hoechst and PI live-dead cell staining results (Figures 8B, C) indicated that after 24 h of incubation with 200 μM of the drug, the survival rate of 266–6 cells exceeded 80%. Subsequently, we extracted primary pancreatic acinar cells and used STC stimulation to establish a *in vitro* model of AP, with interventions of Stigma at concentrations of 50 μM and 100 μM. The results showed that STC stimulation led to a significant increase in cell necrosis, with the proportion of necrotic cells rising from 8.5% in the control group to 69.3% in the model group. Treatment with Stigma significantly reduced the proportion of necrotic cells, with the most pronounced inhibitory effect observed at 100 µM Stigma (Figures 8D, E). This data suggests the potential protective role of Stigma against STC-induced necrosis in pancreatic acinar cells.

### 3.8 Stigma promotes acinar cells apoptosis in STC-induced AP

**FIGURE 9:**
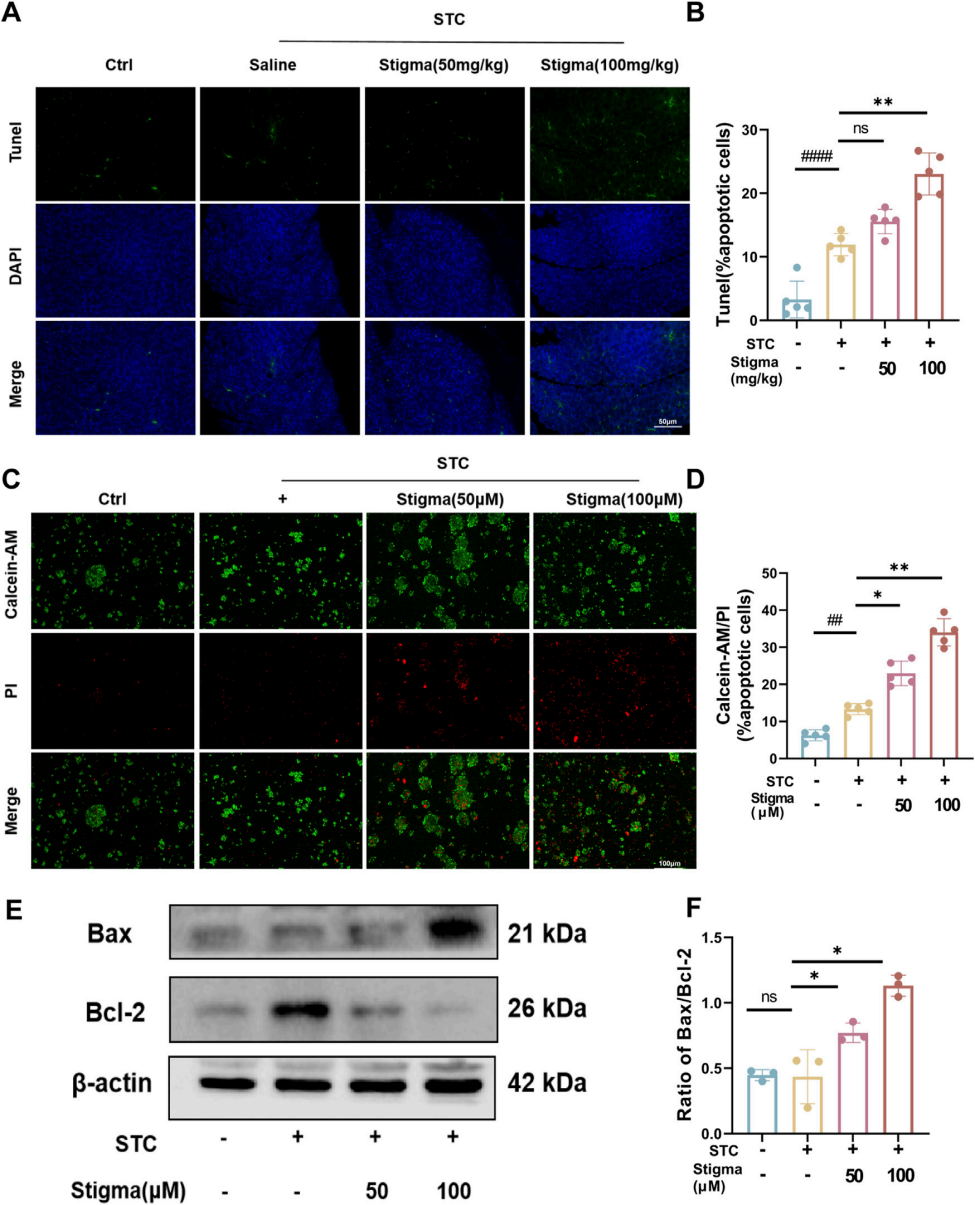
Stigma promotes acinar cell apoptosis in STC-induced AP. **(A)** Representative images of TUNEL staining for the evaluation of apoptotic acinar cells in mice with STC-induced AP 50 μm scale bar. **(B)** Statistical results on the proportion of pancreatic acinar cells undergoing apoptosis in each group. **(C)** Representative images of Calcein-AM/PI staining from pancreatic acinar cells, 100 μm scale bar. **(D)** Statistical results on the proportion of pancreatic acinar cells undergoing apoptosis in each group. **(E, F)** Representative images and quantitative analysis of Bax as well Bcl-2 proteins in STC-stimulated primary pancreatic acinar cells treated with or without Stigma. All data are expressed as mean ± SD. n = 3 or 5. All the *p < 0.05. ns: not significant.

Typically, apoptosis is regarded as advantageous upon the initiation of AP, as it effectively averts the propagation of the inflammatory cascade, thereby mitigating deleterious consequences. TUNEL staining was used to analyze apoptosis of acinar cells, and our results revealed pancreatic acinar cells underwent apoptosis upon the induction of STC, as depicted in Figure 9A. After Stigma treatment, the incidence of TUNEL-positive cells has significantly increased (Figure 9B). Calcein-AM/Propidium Iodide (PI) Staining shows increased number of PI-Positive cells after Stigma treatment (Figures 9C, D). We investigated the expression of Bcl-2 and Bax by WB in acinar cells. The WB analysis demonstrated that Stigma exhibited markedly elevated the ratio of Bcl-2/Bax in comparison to both the sham and AP groups. WB analysis showed that Stigma exhibited a significantly elevated Bcl-2/Bax ratio compared to the sham and AP groups (Figures 9E, F). This indicates that Stigma significantly enhanced the apoptosis of pancreatic acinar cells in AP.

## 4 Discussion

AP is manifested by pancreatic damage and inflammatory responses, leading to local and systemic complications, with high morbidity and mortality worldwide (Tarasiuk and Fichna, 2019; Lodewijkx et al., 2016; Yadav and Lowenfels, 2013). Current treatment guidelines for AP include intravenous fluids, dietary changes, analgesics, pancreatic secretory inhibitors (somatostatin and its analogues octreotide) (Dimagno, 2015). Specific therapeutic modalities, including trypsin inhibition to reduce necrosis of pancreatic tissue, such as the use of ustekinumab with mebeverine and octreotide, have shown limited efficacy. There is a lack of drugs that can significantly inhibit necrosis of pancreatic cells and uncontrolled inflammatory responses. As a result, there is renewed interest in botanicals, which have no serious adverse effects and are beneficial not only for symptoms but also for disease evolution. In addition, dietary preparations such as phytochemicals, which generally remain non-toxic even at relatively high doses and are inexpensive, have great potential. Recent studies have shown that some phytochemical components have been proven to have good pharmacological effects in the treatment of AP, such as lycopene, curcumin, cinnamin B-1, capsaicin, piperine, lycopene, resveratrol (Thorat et al., 1995; Sidhu et al., 2011; Wang et al., 2014; Bae et al., 2011; Ozkan et al., 2012). But so far, there are no studies exploring the therapeutic effect of Stigma on AP. This study represents the first exploration into the role of Stigma in the AP process.

Stigma is a natural sterol that features a double bond at the C-22 position (Mora-Ranjeva et al., 2006). It belongs to a larger class of plant compounds known as phytosterols, which are widely found in plant-based foods and common medicinal plants across the globe (Ryan et al., 2007). Research has shown that Stigma possesses a variety of pharmacological activities, including anti-inflammatory and anti-oxidative stress properties (Antwi et al., 2017; Feng et al., 2017; Morgan et al., 2021; Sampath et al., 2021a). Specifically, Liang et al. discovered that Stigma could reduce the production of free radicals and lipid peroxidation, demonstrating its effectiveness in combating oxidative damage (Sampath et al., 2021b). Fan J et al. demonstrated that Stigma could mediate the secretion of inflammatory cytokines by regulating NF-κB signaling or the NLRP3 inflammatome (Feng et al., 2017). Liang et al. found that Stigma can relieve oxidative stress, inflammation, and apoptotic responses to a certain extent to protect the brain from brain I/R damage (Liang et al., 2020). However, the anti-inflammatory effects of Stigma have yet to be studied in the context of the STC-induced AP model. Our study demonstrates that, compared to existing treatments for AP, Stigma significantly reduces acinar cell necrosis and inflammatory factor release, inhibits the secretion of amylase and lipase in experimental pancreatitis models *in vitro* and *in vivo*, providing a substantial anti-pancreatitis effect. Additionally, its analog, the isospirostanol analog diosgenin, has therapeutic effects on AP (Zhang et al., 2016). However, diosgenin has cell perforation, hemolysis and hormone-like side effects such as gonadotropin hyperplasia and reproductive toxicity, which seriously limits its clinical application (Lin and Wang, 2010; Gao et al., 2024). While, Stigma exhibits no such side effects and has weaker hormone-like effects during the anti-inflammatory process, making it a more advantageous candidate as an anti-pancreatitis drug.

Network pharmacology is a powerful approach for identifying bioactive compounds and predicting drug targets using advanced computational simulations. Through molecular docking studies, we have identified that Stigma exhibits the strongest binding affinity with ERK1, suggesting that ERK1 is a promising target for Stigma. Furthermore, and the interaction between Stigma and ERK1 was directly verified by MST, which combined with WB and IHC to detect the phosphorylation level of ERK1, providing evidence that Stigma is actively involved in regulating the activation of ERK1 signaling pathway.

The MAPK signaling pathway plays a central role in regulating cellular behavior and the signaling of inflammatory factors, thereby influencing immune cell function and enhancing the activation of the immune system to combat foreign pathogens. This pathway also plays a key role in modulating and reducing the body’s inflammatory response (Zhou et al., 2020; Ma et al., 2018). Among the MAPK family members, ERK1 (MAPK3) is particularly significant in controlling inflammatory responses and apoptotic pathways. The Raf/MEK/ERK1 axis represents a classic signaling cascade within the MAPK subfamily. Phosphorylated Raf (p-Raf), the initiator of this pathway, triggers the phosphorylation of downstream MEK (p-MEK), which in turn activates and phosphorylates ERK1 (p-ERK), thereby influencing the expression of downstream inflammatory mediators. This cascade ultimately modulates the overall inflammatory response (He et al., 2024). By integrating network pharmacology with previous research findings, we analyzed the expression and mRNA levels of the ERK1 pathway-associated proteins RAS, RAF, and ERK1 in mouse acinar cells. The results from qPCR, WB, and IHC revealed a significant downregulation of ERK1, KRAS, and B-RAF mRNA and protein expression levels compared to the model group. These findings suggest that Stigma may alleviate AP inflammation by promoting apoptosis and inhibiting ERK1 expression.

Apoptosis, initially defined as a physiological or programmed form of cell death, is characterized by distinct morphological changes such as cell atrophy, retention of organelles, and nuclear chromatin condensation, all of which occur in response to various stress-related stimuli. The regulation of apoptosis is primarily governed by the Bax and Bcl-2 genes, with the Bax/Bcl-2 ratio, rather than the absolute levels of these proteins, serving as a critical determinant of a cell’s susceptibility to apoptosis (Hou et al., 2017; Vucicevic et al., 2016). The present study demonstrated that the therapeutic mechanism of Stigma in treating AP was closely linked to ERK1, which plays a pivotal role in regulating the expression of apoptosis-related molecules such as Bcl-2 and Bcl-XL. These molecules are essential for cell growth, development, and proliferation (Chang et al., 2003). ERK1 activation can upregulate anti-apoptotic proteins like Bcl-2 and induce its activation, which in turn promotes Bcl-2 expression, inhibits cytochrome c (Cyt-c) release, preserves mitochondrial function, and suppresses apoptosis (Romerio and Zella, 2002). Additionally, ERK1 can inhibit the pro-apoptotic activity of Bim by preventing its binding to Bax through phosphorylation (Harada et al., 2004). Recent studies showed that the degree of apoptosis in pancreatic acinar cells was inversely correlated with the severity of acute pancreatitis, suggesting that apoptosis may serve as a protective mechanism in this condition (Fuchs and Steller, 2011; Bhatia, 2004). In this study, apoptosis was assessed using TUNEL and Calcein-AM/PI staining, and the results showed that Stigma treatment promoted apoptosis. Specifically, we observed an increase in Bax expression and a decrease in Bcl-2 expression, leading to a significant elevation in the Bax/Bcl-2 ratio. These findings suggest that Stigma-induced cell death is regulated by the Bax/Bcl-2 pathway.

The comprehensive approach employed in this study establishes a solid foundation for further exploration of natural product components, their associated targets, and potential mechanisms of action in the treatment of AP. While these findings offer valuable insights into the therapeutic potential of Stigma for treating AP, there are still a series of defects. First, our research model STC-induced AP is to simulate cholestatic pancreatitis (Chen et al., 2018), in addition to cholestatic pancreatitis, there are other types of clinical AP, such as alcohol-origin pancreatitis, hypertrig lyceridemia-AP, etc., whether Stigma can protect against other types of remains to be further studied. Meanwhile, the STC-induced pancreatitis model is widely regarded as closely mimicking SAP due to the significant tissue damage and systemic inflammatory response it generates. While, this model is unsuitable for studying mild pancreatitis, which presents with less severe symptoms. In addition, STC-AP is still different from real clinical cholestasis and cannot completely replicate human disease, and the AP-related mechanisms discovered based on these modeling studies must be interpreted with caution, and the future translation of Stigma still needs clinical support. Network pharmacology utilizes computer simulations and various databases to screen drug molecular targets and predict their signaling pathways and mechanisms of action, but the network pharmacology approach still has inherent limitations in predicting *in vivo* outcomes. To compensate for these shortcomings, we first used MST to provide direct evidence for the binding of Stigma and ERK1, and then our *in vitro* and *in vivo* experimental results further solidified the effect of Stigma on the ERK1 signaling pathway. Additional fundamental research and clinical trials are essential to validate these conclusions and address any safety concerns related to Stigma.

## 5 Conclusion

This study represents the first attempt to integrate network pharmacology with experimental validation to explore the mechanism of Stigma in treating AP. The results confirmed that Stigma can mitigate the severity of AP by inhibiting the RAS/RAF/MEK/ERK1 signaling pathway and promoting the apoptosis of acinar cells. These findings suggest that Stigma could be a promising therapeutic agent for AP, offering a novel and effective approach to managing this condition.

## Data Availability

The raw data supporting the conclusions of this article will be made available by the authors, without undue reservation.
