# Supplementary material for: Elucidating the mechanism of stigmasterol in acute pancreatitis treatment: insights from network pharmacology and in vitro/in vivo experiments
Source: Front Pharmacol. 2024 Dec 23;15:1485915. doi: 10.3389/fphar.2024.1485915 (PMC11701227; doi:10.3389/fphar.2024.1485915)
Supplement: Supplementary file 1 [file DataSheet1.pdf]

Elucidating the Mechanism of Stigmasterol in Acute Pancreatitis Treatment: Insights from Network Pharmacology and In Vitro/In Vivo Experiments

ONLINE SUPPLEMENTARY MATERIAL

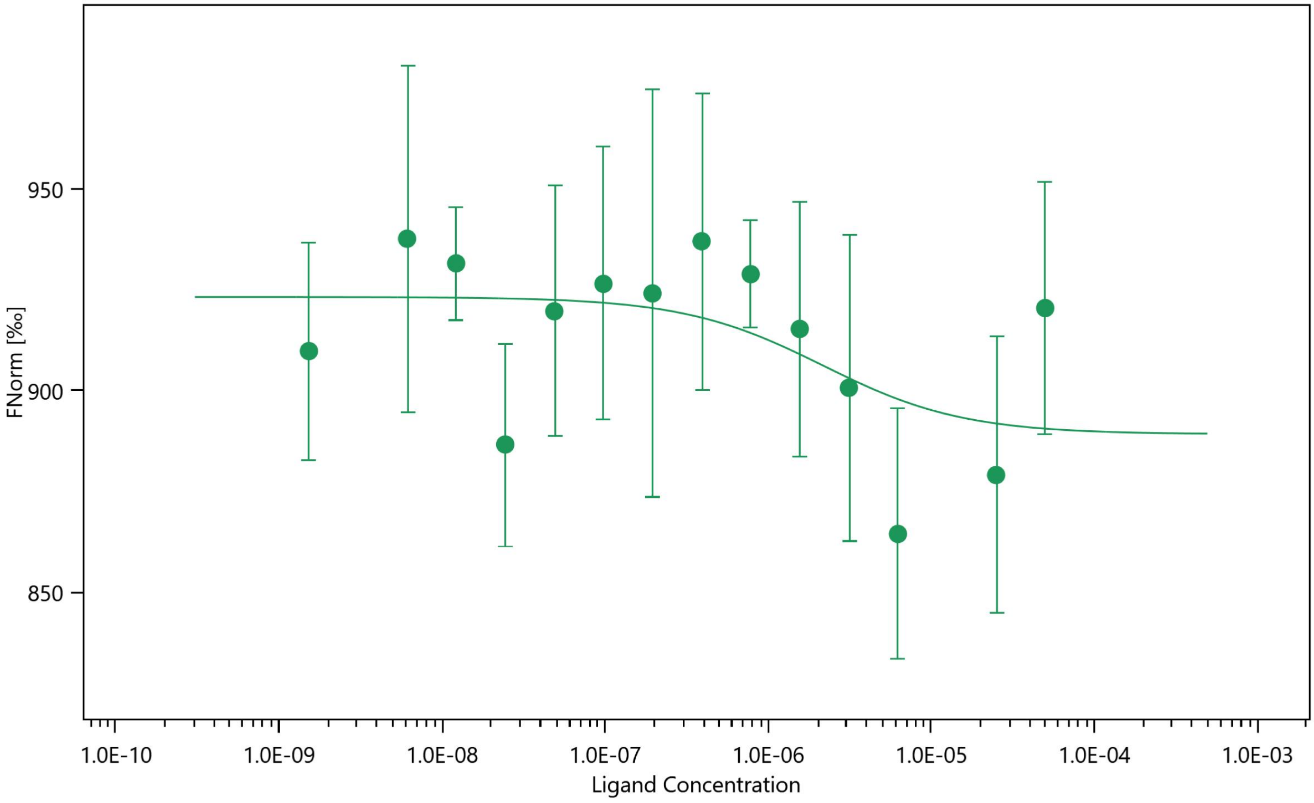

Figure S1 MST data analysis. Plot of the normalized fluorescence fraction bound vs. the concentration of ERK1 from MST experiments. Lines represent fits of the data points using the Kd equation.
